# Evaluation of the data-collection strategy for room-temperature micro-crystallography studied by serial synchrotron rotation crystallography combined with the humid air and glue-coating method

**DOI:** 10.1107/S2059798321001686

**Published:** 2021-02-25

**Authors:** Kazuya Hasegawa, Seiki Baba, Takashi Kawamura, Masaki Yamamoto, Takashi Kumasaka

**Affiliations:** a Protein Crystal Analysis Division, Japan Synchrotron Radiation Research Institute, 1-1-1 Kouto, Sayo-cho, Sayo-gun, Hyogo 679-5198, Japan; b Advanced Photon Technology Division, RIKEN SPring-8 Center, 1-1-1 Kouto, Sayo-cho, Sayo-gun, Hyogo 679-5148, Japan

**Keywords:** serial synchrotron crystallography, room-temperature data collection, humidity control, radiation damage, macromolecular crystallography

## Abstract

Synchrotron serial crystallography (SSX) is an emerging data-collection method for micro-crystallography on synchrotron macromolecular (MX) crystallography beamlines. At SPring-8, the feasibility of the fixed-target approach was examined by collecting data using a 2D raster scan combined with goniometer rotation. Results at cryogenic temperatures demonstrated that rotation is effective for efficient data collection in SSX and the method was named serial synchrotron rotation crystallography (SS-ROX). To use this method for room-temperature (RT) data collection, a humid air and glue-coating (HAG) method was developed in which data were collected from polyvinyl alcohol-coated microcrystals fixed on a loop under humidity-controlled air. The performance and the RT data-collection strategy for micro-crystallography were evaluated using microcrystals of lysozyme. Although a change in unit-cell dimensions of up to 1% was observed during data collection, the impact on data quality was marginal. A comparison of data obtained at various absorbed doses revealed that absorbed doses of up to 210 kGy were tolerable in both global and local damage. Although this limits the number of photons deposited on each crystal, increasing the number of merged images improved the resolution. On the basis of these results, an equation was proposed that relates the achievable resolution to the total photon flux used to obtain a data set.

## Abstract 2

A new room-temperature (RT) data-collection method for microcrystals was developed by combining serial synchrotron rotation crystallography with the humid air and glue-coating method. The RT data-collection strategy for micro-crystallography was evaluated by examining the efficiency, the influence of non-isomorphism and radiation damage, and the effectiveness of increasing the number of merged images. An equation was proposed that relates the achievable resolution to the total photon flux used to obtain a data set.

## 1. Introduction   

Cryocrystallography is an essential technique to maximize the benefits of the high-brilliance beams available at synchrotron facilities by mitigating radiation damage (Garman & Owen, 2006 ▸). In the past two decades, most macromolecular crystallography (MX) data collection has been conducted at cryogenic temperatures (CT), contributing to the rapid growth in the number of protein structure determinations (Pflugrath, 2015 ▸). Together with the development of microfocus beamlines at synchrotron facilities, cryocrystallography has enabled the successful structure determination of challenging targets such as membrane proteins, large and high-quality crystals of which are difficult to prepare (Yamamoto *et al.*, 2017 ▸). However, the importance of structure determination at room temperature (RT) has been revisited because it has been shown that cryocooling can hide structures that have biological significance (Fraser *et al.*, 2009 ▸, 2011 ▸; Fischer *et al.*, 2015 ▸).

The difficulty in RT data collection is radiation damage, which appears at absorbed doses two orders of magnitude lower compared with that at CT (Helliwell, 1988 ▸; Nave & Garman, 2005 ▸; Southworth-Davies *et al.*, 2007 ▸) and means that the amount of data collected from one crystal is reduced by two orders of magnitude. Therefore, data collection from multiple crystals is needed for RT data collection when the completion of a data set is difficult using a single crystal; this is quite usual in the case of micro-crystallography. In the case of CT, a useful data-collection method is multiple small-wedge data collection, in which data are collected from each crystal using an angular range of a few degrees to 10° and are merged after data collection. By combining this method with a 2D raster scan to identify the position of the crystal on the diffraction base, data collection can be automated. This makes it easy to use hundreds of crystals or more to complete the data set (Hirata *et al.*, 2019 ▸). However, an alternative efficient method is needed for RT data collection because the number of crystals needed to complete the data set is larger and the 2D raster scan prior to data collection needs to be avoided.

Serial femtosecond crystallography (SFX), which was developed at X-ray free-electron lasers (XFELs), has introduced a new approach in MX data collection, *i.e.* the collection of thousands to hundreds of thousands of single-shot still images by irradiating a crystal using femtosecond X-ray pulses (Chapman *et al.*, 2011 ▸; Schlichting, 2015 ▸). Crystals are delivered into an X-ray beam either by a continuous flow of crystal suspension from a liquid injector (injector-based SFX) or by the translation of a sample holder onto which microcrystals are loaded (fixed-target SFX). The integrated intensities extracted from each indexed image are merged by the Monte Carlo integration method (Kirian *et al.*, 2010 ▸; White *et al.*, 2016 ▸), yielding complete data with sufficient accuracy for structure determination. Based on the principle of ‘diffraction before destruction’ (Neutze *et al.*, 2000 ▸), SFX enables structure determination at RT without radiation damage. The usefulness of SFX has been demonstrated particularly well in a time-resolved structural study (Schmidt, 2019 ▸). The success of SFX led to the development of synchrotron serial crystallography (SSX) using either a continuous flow of a crystal suspension (see, for example, Stellato *et al.*, 2014 ▸; Botha *et al.*, 2015 ▸; Nogly *et al.*, 2015 ▸; Martin-Garcia *et al.*, 2017 ▸; Weinert *et al.*, 2017 ▸) or a fixed-target method (see, for example, Gati *et al.*, 2014 ▸; Coquelle *et al.*, 2015 ▸; Owen *et al.*, 2017 ▸; Wierman *et al.*, 2019 ▸). Even though radiation damage must be taken into consideration, the feasibility of SSX has been demonstrated and it has successfully been applied to time-resolved structure analysis (Schulz *et al.*, 2018 ▸; Weinert *et al.*, 2019 ▸; Aumonier *et al.*, 2020 ▸). Moreover, efficient data collection using a pink beam has been demonstrated (Meents *et al.*, 2017 ▸; Tolstikova *et al.*, 2019 ▸; Martin-Garcia *et al.*, 2019 ▸). At SPring-8, we examined the feasibility of a fixed-target approach following the protocol of Gati and coworkers, in which data are collected using a 2D raster scan combined with goniometer rotation (Gati *et al.*, 2014 ▸). Our results obtained at CT demonstrated that rotation is effective for efficient data collection in SSX and we called this method serial synchrotron rotation crystallography (SS-ROX; Hasegawa *et al.*, 2017 ▸). Meanwhile, Wierman and coworkers reported the performance of serial oscillation crystallography with a fixed target, which demonstrated that a larger oscillation wedge decreased the total number of crystals needed to complete a data set (Wierman *et al.*, 2019 ▸).

The essential technique for RT SSX is to maintain sample quality during data collection. In the case of an injector-based method, samples are kept in the mother solution or in high-viscosity media. In the case of a fixed-target method, two approaches are possible: one is sealing the sample holder with a polymer film (Owen *et al.*, 2017 ▸; Wierman *et al.*, 2019 ▸) and the other is the use of a humidifier (Roedig *et al.*, 2016 ▸; Tolstikova *et al.*, 2019 ▸).

We developed the humid air and glue-coating (HAG) method (Baba *et al.*, 2013 ▸) as a method for post-crystallization treatment and RT data collection. Before the development of the HAG method, the use of a humidifier for MX data collection had been already reported as a post-crystallization treatment method to improve the crystal quality by using a capillary-free mounting system (Kiefersauer *et al.*, 2000 ▸) or a humidity-control device (Sanchez-Weatherby *et al.*, 2009 ▸). The big difference between the HAG method and these methods is that the HAG method coats crystals with a glue, polyvinyl alcohol (PVA), which provides the advantage that the environment of the crystals can be changed without losing crystal quality and makes cooling after optimization of humidity easy (Baba *et al.*, 2013 ▸). Based on the usefulness of PVA coating, we have developed a technique to control the temperature down to 4°C using the HAG method (Baba *et al.*, 2019 ▸), leading to successful RT data collection for cytochrome *c* oxidase crystallized at 4°C (Shimada *et al.*, 2017 ▸).

In this study, we have used the HAG method to perform SS-ROX data collection and demonstrated that it is applicable to RT micro-crystallography. We also evaluated the RT data-collection strategy for micro-crystallography by examining the efficiency, the influence of non-isomorphism and radiation damage, and the effectiveness of increasing the number of merged images.

## 2. Experimental   

### 2.1. Preparation of microcrystals   

Hen egg-white lysozyme microcrystals were prepared following the protocols of Falkner *et al.* (2005 ▸) and Nango *et al.* (2015 ▸) with some modifications. Lysozyme powder (catalogue No. L6876-5G, Lot No. SLBT5180, Sigma–Aldrich) was dissolved in 10 m*M* sodium acetate pH 4.6 to a concentration of 40 mg ml^−1^. The lysozyme from this lot has a property to generate numerous microcrystals when used in crystallization. 100 µl lysozyme solution was mixed with the same volume of 4 *M* sodium malonate pH 3.1, 6%(*w*/*v*) PEG 6000 and stirred for 20 min at 20°C using a ThermoMixer C (Eppendorf). Crystal growth was stopped by adding 800 µl 2.4 *M* sodium malonate pH 3.1, 3.6%(*w*/*v*) PEG 6000 to decrease the lysozyme concentration. The size of the crystals can be controlled by changing the time of crystal growth. The suspension of microcrystals was then spun down at 3000*g*. After the removal of the supernatant, 2.4 *M* sodium malonate pH 3.1 was added to resuspend the microcrystals at a concentration of 4 × 10^7^ ml^−1^, which was measured by counting the number of crystals using a cell-counter plate (catalogue No. 177-112C, Watson Bio Lab) under a stereomicroscope. Microcrystals of around 15 µm were used in this study to collect diffraction data to better than 2 Å resolution to enable the detection of small structural changes caused by local radiation damage.

### 2.2. Sample mounting   

**Figure 1:**
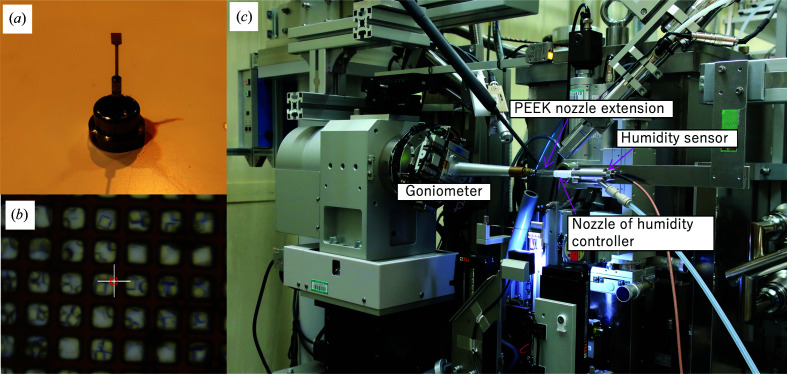
(*a*) 2 × 2 mm square mesh-loop attached to the goniometer base. (*b*) Crystals loaded onto the mesh-loop observed by an on-axis camera. The crystals were coated with PVA. The white cross at the center corresponds to 50 µm and the red rectangle represents a beam size of 10 × 8.7 µm (vertical × horizontal; FWHM). (*c*) Setup of the HAG SS-ROX experiment on BL41XU at SPring-8.

The lysozyme microcrystals were loaded onto a specially designed square polyimide mesh-loop with a size of 2 × 2 mm that had regularly arranged windows of 40 × 40 µm with a 10 µm line of polyimide between them (Protein Wave Corporation; Figs. 1 ▸ *a* and 1 ▸ *b*). The thickness of the mesh was 25 µm. After spreading 0.25 µl of crystal suspension over the mesh-loop using a pipette, 10%(*w*/*v*) PVA with an average polymerization degree of 4500 containing 5%(*v*/*v*) glycerol was spread from the opposite side of the mesh-loop using a toothpick. During this process, a substantial amount of crystal suspension was extruded to the back side through the mesh windows. The excess crystal suspension was removed together with the excess PVA. After mounting the mesh-loop on the goniometer, air with a relative humidity (RH) of 73% was blown from the spindle direction at a flow rate of 8 ml min^−1^. Before equilibrium under the humidity-controlled air, the PVA solution has sufficient fluidity that the crystals diffuse into the PVA. We observed that the microcrystals were coated by the PVA glue using an optical microscope, as shown in Supplementary Fig. S1. The humidity controller used in this study was a HUM1-F (Rigaku). The temperature of the humidity-controlled air was determined by the temperature of the experimental hutch, which was controlled at 25°C. To stabilize the air flow and maintain constant humidity around the crystal, the mesh-loop was surrounded by a polyether ether ketone (PEEK) film extending from the nozzle of the humidity blower (Fig. 1 ▸ *c*). The thickness of the PEEK film was 12 µm. A side view of the mesh-loop onto which sample was loaded is shown in Supplementary Fig. S2, showing that the thickness of the PVA is marginal.

### 2.3. Data collection and data processing   

Data collection was performed on BL41XU at SPring-8 (Hasegawa *et al.*, 2013 ▸) using a wavelength of 1 Å. The beam size was 10 × 8.7 µm (vertical × horizontal; FWHM) and the photon flux without attenuation was 4.8 × 10^12^ photons s^−1^. The beam profile deviated from a Gaussian profile because the horizontal beam size was shaped using a slit at the secondary source, and the vertical beam size of 10 µm was obtained by changing the glancing angle of the focusing mirror from the best focus. The detector was an EIGER X 16M (Dectris) with a camera distance of 180 mm. Diffraction data were collected by SS-ROX; 100 horizontal helical scans were performed at 20 µm intervals in the vertical direction. In each helical scan, 222 images were collected with a translation step of 9 µm per image and a rotation step of 0.25° per image. A total of 14 data sets were collected using seven different dose conditions, 21, 42, 83, 210, 420, 830 and 1700 kGy, to inspect the influence of radiation damage (Table 1 ▸). These data are referred to in the following as L*XX*k-1 and L*XX*k-2 for the first and second data sets, respectively, where *XX* represents the dose in kGy. The dose was adjusted by combining the frame rate of the detector and attenuation of the incident beam intensity, which resulted in the use of different dose rates except for the data sets L420k, L830k and L1700k (Table 1 ▸). The dose was estimated using *RADDOSE*-3*D* (Zeldin, Gerstel *et al.*, 2013 ▸), assuming a uniform beam profile of 10 × 8.7 µm (vertical × horizontal; FWHM) and crystal dimensions of 15 × 15 × 15 µm. The condition in which the beam completely passed through a crystal was used to estimate the dose in the helical scan. The average dose-exposed region (Zeldin, Brockhauser *et al.*, 2013 ▸) was used as a metric for the absorbed dose.

The contribution of background scattering from the PEEK film, PVA and polyimide of the mesh-loop was evaluated by collecting four scattering images: (i) without the PEEK film and the mesh-loop, (ii) with the PEEK film without mounting the mesh-loop, (iii) with the PEEK film and a PVA-coated mesh-loop by illuminating with X-rays at the center of a window of the mesh-loop and (iv) with the PEEK film and a PVA-coated mesh-loop by illuminating with X-rays at the polyimide of the mesh. All data were collected with a 1 s exposure time and an oscillation of 0.25° using a 9.8 × 9.4 µm (vertical × horizontal) beam with a photon flux of 5.6 × 10^12^ photons s^−1^ and a camera distance of 180 mm.

Hit images were identified using the spot-finding software *SHIKA* (Hirata *et al.*, 2019 ▸), which uses *Cheetah* (Barty *et al.*, 2014 ▸) to find diffraction spots in each image. Images containing more than three spots within 5 Å resolution were assigned as hit images. Hit images were indexed and integrated with *indexamajig* from *CrystFEL* version 0.8.0 (White *et al.*, 2016 ▸) using *DirAx* (Duisenberg, 1992 ▸) for indexing. After applying the Lorentz factor correction using the script correct_stream_nonempirical.py (https://github.com/keitaroyam/yamtbx), *CrystFEL* streams were merged using *process_hkl* from *CrystFEL* version 0.7.0 with per-image linear scaling.

Lysozyme structures were refined from an initial model which was prepared as follows: after all heteroatoms had been removed from the lysozyme structure determined at RT (PDB entry 4eta; Boutet *et al.*, 2012 ▸), it was refined against data derived by merging 9000 images collected at 83 kGy. Two malonate ions, one sodium ion and 49 waters were incorporated into the structure during this refinement. No further waters or malonate ions were added, and no alternate conformations were modeled during refinement of the structures shown in Table 2 ▸. *Phenix.refine* (Afonine *et al.*, 2012 ▸) and *Coot* (Emsley *et al.*, 2010 ▸) were used for refinement and model building, respectively.

The resolution of each data set was estimated by a CC_1/2_ versus resolution plot by fitting the function ½{1 − tanh[(*s* − *d*_0_)/*r*]} × dcc − dcc + *b* that is used in *AIMLESS* (http://www.ccp4.ac.uk/html/aimless.html) in the *CCP*4 package (Winn *et al.*, 2011 ▸), where *s* denotes the square inverse of the Bragg spacing and the other variables *d*_0_, *r*, dcc and *b* are determined by least squares. In the original function in *AIMLESS* *b* is fixed at 1, whereas we introduced it as a variable because a number of the data sets, especially those prepared by merging small numbers of images, had a CC_1/2_ of less than 1 even in low-resolution shells.

The root-mean-square difference (r.m.s.d.) between the two structures was calculated by *LSQKAB* (Kabsch, 1976 ▸) in the *CCP*4 package. The Wilson *B* factor (*B*_Wilson_) was calculated by *phenix.xtriage* (Zwart *et al.*, 2005 ▸). The average *B* factors were calculated by *MOLEMAN* (Kleywegt *et al.*, 2001 ▸). The molecular-graphics figures were prepared using *PyMOL* (version 2.3; Schrödinger).

## 3. Results and discussion   

### 3.1. Performance of HAG SS-ROX   

The HAG method has effectively been applied to various protein crystals (Baba *et al.*, 2013 ▸). Here, we examined the application of the method to protein microcrystals. As shown in Supplementary Fig. S1, we confirmed that the microcrystals were spontaneously immersed into PVA glue within a few minutes and were not exposed to the humid air. The glue thickness was roughly estimated as a few tens of micrometres by microscopic depth measurements. As described in the previous report (Baba *et al.*, 2013 ▸), larger crystals could be thoroughly coated and covered by the glue. This observation is quite similar to the present result. This suggests that the coating mechanism includes the effect of wetting. Indeed, when we observed the interface between PVA and a crystal suspension deposited side by side between two glass plates, the liquid of the crystal suspension dissolved PVA from the glue and a new phase, which was a mixture of liquid and glue, appeared at the interface. The liquid phase is also condensed under the humid air, which finally led to coating of the crystals with the glue while the crystals were kept wetted.

The influence of the background scattering from the PEEK film, PVA and polyimide of the mesh-loop is shown in Supplementary Fig. S3, which shows that the increase in background scattering caused by coating with PVA is only a few percent. However, the PEEK film and the polyimide of the mesh-loop show a significant increase in background scattering, especially at 4.8 Å resolution, where both of the polymeric materials have a broad peak.

**Figure 2:**
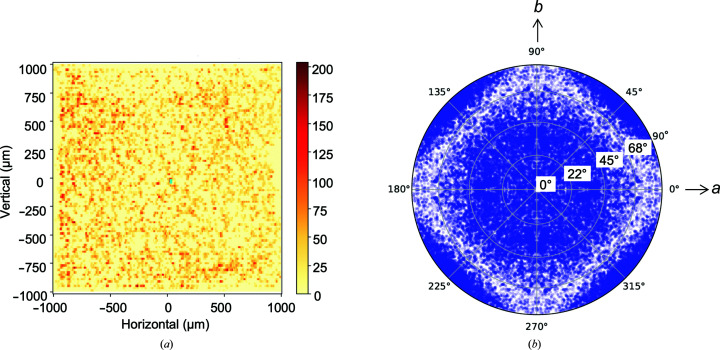
(*a*) Heat map showing the distribution of crystals on the mesh-loop for L83k-2. The number of spots found up to 5 Å resolution is indicated in accordance with the color table shown on the right. (*b*) Distribution of crystal orientation. Each blue dot indicates the direction of the incident beam relative to the unit cell. The right and top directions correspond to the *a* and *b* axes, respectively. The *c* axis is directed towards the reader. 422 symmetry was imposed during calculation.

The distribution of the crystals in the mesh-loops was identified and illustrated by *SHIKA* as a heat map (shown in Fig. 2 ▸ *a*). The crystals were evenly distributed over the mesh-loop. It also shows the crystals were arranged in a 2D lattice, reflecting that many of them were trapped in the windows of the mesh-loop, as shown in Fig. 1 ▸(*b*). The distribution of the incident beam direction relative to the unit-cell axes is illustrated in Fig. 2 ▸(*b*), showing a tendency for the X-rays to hit along the median line between the *a* and *b* axes or along the *c* axis. This indicates that the crystals were fixed with their flat surface parallel to the mesh-loop. The problem caused by this preferred orientation might have been mitigated by rotation of the mesh-loop by ±27.25° during the helical scan.

The hit rate and index rate are summarized in Table 1 ▸. The maximum hit rate was 69.0% for L830k-2 and the minimum hit rate was 15.6% for L1700k-2. There was no clear dose-dependence. The fluctuations in the hit rate are caused by the reproducibility of the total amount of crystals loaded on the loop. The maximum index rate was 82.1% for L83k-2 and the minimum index rate was 44.9% for L830k-2. A lower hit rate tends to yield a higher index rate, implying that a high hit rate results in the failure of indexing of a number of images caused by multiple hits. The overall hit rate of the 14 data sets was 41.9%, and 66.9% of them were successfully indexed.

**Figure 3:**
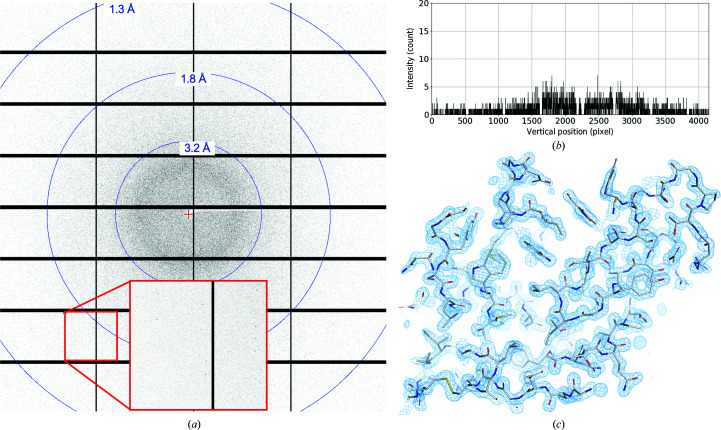
(*a*) Diffraction image recorded at 42 kGy. (*b*) Background profile of the diffraction image shown in (*a*). (*c*) Stick model refined at 1.7 Å resolution using 9000 images of L42k-2 together with a 2*mF*_o_ − *DF*_c_ electron-density map contoured at 1.5σ (sky blue). O, N and S atoms in the model are shown in red, blue and yellow, respectively.

A diffraction image recorded at 42 kGy is shown in Fig. 3 ▸(*a*) together with the background profile (Fig. 3 ▸ *b*). To evaluate the quality of a data set obtained by this method, 9000 images of L42k-2 from 9548 indexed images were merged as described in the next section (data set D_42k-2_9000_). Table 2 ▸ shows that 〈*I*/σ(*I*)〉 and CC_1/2_ in the highest resolution shell 1.73–1.70 Å were 2.18 and 0.71, respectively, with a completeness of 100% and a multiplicity of 253.8. The structure was well refined, with a final *R*_free_ of 21.07% and *R*_work_ of 18.29% and good stereochemistry (Table 2 ▸). The 2*mF*_o_ − *DF*_c_ electron-density map in Fig. 3 ▸(*c*) shows the features of structural analysis at 1.7 Å resolution.

### 3.2. Influence of non-isomorphism of crystals   

**Figure 4:**
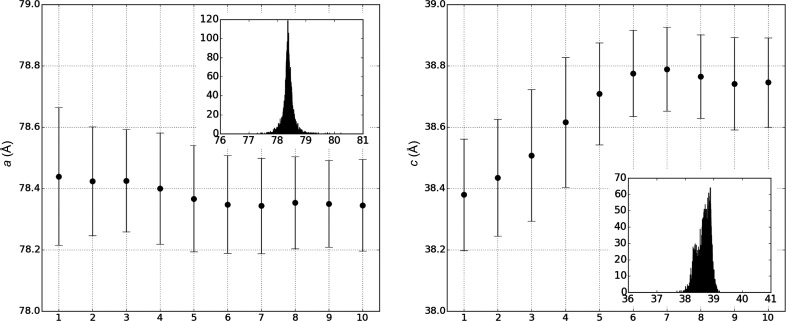
Change in unit-cell dimensions during data collection for L42k-2. After 22 200 images, including non-hit images, had been divided into ten bins in sequential order, average unit-cell dimensions for the indexed images were calculated for each bin and shown as a function of bin number. The error bar corresponds to ±σ. Inset: a histogram showing the distribution of unit-cell dimensions for the 9548 images of L42k-2. The histogram was prepared by a homemade script using the output stream of *CrystFEL*.

**Figure 5:**
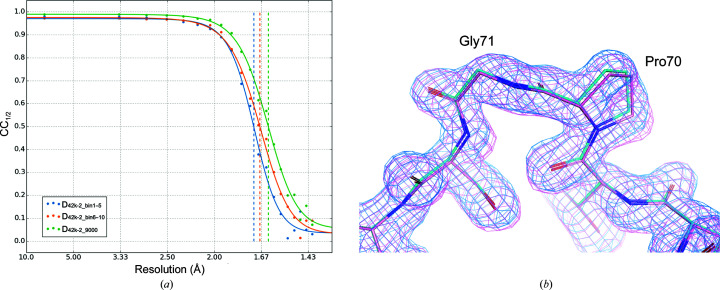
(*a*) CC_1/2_ of D_42k-2_bin1–5_ (sky blue), D_42k-2_bin6–10_ (orange) and D_42k-2_9000_ (green) as a function of resolution together with a fitted line. (*b*) Stick model and 2*mF*_o_ − *DF*_c_ electron-density map calculated at 1.7 Å resolution and contoured at 1σ. C atoms and electron density for D_42k-2_bin1–5_ are shown in cyan and those for D_42k-2_bin6–10_ are shown in pink. O and N atoms in both models are shown in red and blue, respectively.

During the data processing of L42k-2, we noticed a gradual change in unit-cell dimensions during data collection, as shown in Fig. 4 ▸, *i.e.* *a* decreased from 78.44 to 78.34 Å, whereas *c* increased from 38.38 to 38.79 Å, corresponding to an increase of 1%. The influence of this non-isomorphism was analyzed by preparing data sets D_42k-2_bin1–5_ and D_42k-2_bin6–10_ (Table 2 ▸). D_42k-2_bin1–5_ was prepared using the first 4500 indexed images in bins 1 and 5 in Fig. 4 ▸, where the *c* axis changed gradually from 38.38 to 38.71 Å. D_42k-2_bin6–10_ was prepared using the first 4500 indexed images in bins 6–10, where the unit-cell dimension was almost constant at around 38.76 Å. D_42k-2_9000_ mentioned in the previous section was prepared by merging the indexed images in D_42k-2_bin1–5_ and D_42k-2_bin6–10_. The resolutions at which CC_1/2_ falls to 0.5 were 1.71 Å for D_42k-2_bin1–5_ and 1.68 Å for D_42k-2_bin6–10_, whereas that for D_42k-2_9000_ was 1.63 Å (Fig. 5 ▸ *a*). This result indicates that the resolution was improved by increasing the number of images, even if the length of the *c* axis changes by 1%. Here, caution is needed in the comparison of D_42k-2_bin1–5_ and D_42k-2_bin6–10_, as we noticed that the CC_1/2_ of the data set derived from bins 1–2 was worse than those derived from the other bins (data not shown). Therefore, the lower resolution of D_42k-2_bin1–5_ was mainly due to the incorporation of these inferior quality data. This was confirmed by comparing the difference in achieved resolution between data sets prepared from bins 3–6 and bins 7–10 in Fig. 4 ▸, which shows that the influence of non-isomorphism among these bins was marginal (Supplementary Fig. S4).

To investigate the structural aspects of the non-isomorphism, we calculated the r.m.s.d. among the main chains of the structures refined using these three data sets (Supplementary Fig. S5). The largest r.m.s.d. was observed for Pro70 and Gly71. These two residues interact with Gly71 and Pro70 of a symmetry-related molecule, respectively, by van der Waals interactions. The r.m.s.d.s between these residues for the D_42k-2_bin1–5_ and D_42k-2_bin6–10_ data sets were 0.215 and 0.251 Å, respectively. Stick models and 2*mF*_o_ − *DF*_c_ electron-density maps of D_42k-2_bin1–5_ and D_42k-2_bin6–10_ are shown in Fig. 5 ▸(*b*), which clearly shows a difference in the structures.

A change in unit-cell dimensions during data collection was also observed for data other than L42k-2, as shown in Supplementary Fig. S6.

### 3.3. Influence of radiation damage   

**Figure 6:**
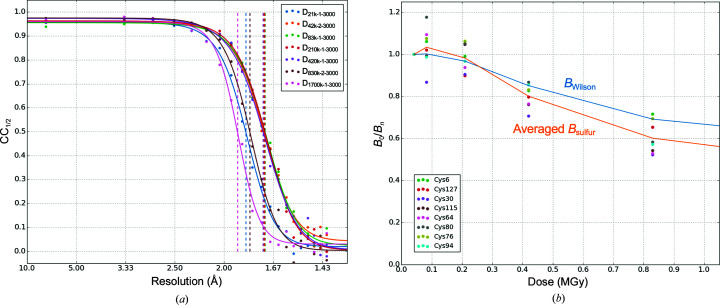
(*a*) CC_1/2_ of D_21k-1-3000_, D_42k-2-3000_, D_83k-1-3000_, D_210k-1-3000_, D_420k-1-3000_, D_830k-2-3000_ and D_1700k-1-3000_ as a function of resolution together with a fitted line. (*b*) *B*_0_/*B*_*n*_ as a function of dose, where *B*_0_ denotes *B*_Wilson_ or *B*_sulfur_ of D_42k-2-3000_ and *B*_*n*_ denotes that of D_83k-1-3000_, …, D_1700k-1-3000_. *B*_0_/*B*_*n*_ for *B*_Wilson_ is shown as a solid sky blue line and *B*_0_/*B*_*n*_ for the averaged *B*_sulfur_ of eight cysteine S^γ^ atoms is shown as a solid orange line. D_21k-1-3000_ was omitted from the plot.

In order to examine the influence of radiation damage, seven data sets were prepared by merging 3000 indexed images. To avoid the influence of non-isomorphism, images in a region where changes in unit-cell dimensions were small were selected from L21k-1, L42k-2, L83k-1, L210k-1, L420k-1, L830-2 and L1700k-1, as shown with two-directional arrows in Supplementary Fig. S6. This limited the number of merged images to 3000. Hereafter, these data sets are referred to as D_21k-1-3000_, D_42k-2-3000_, D_83k-1-3000_, D_210k-1-3000_, D_420k-1-3000_, D_830k-2-3000_ and D_1700k-1-3000_, respectively The resolutions where CC_1/2_ fell to 0.5 were as follows; D_21k-1–3000_, 1.84 Å; D_42k-2-3000_, 1.72 Å; D_83k-1-3000_, 1.72 Å; D_210k-1-3000_, 1.72 Å; D_420k-1-3000_, 1.73 Å; D_830k-2-3000_, 1.81 Å; D_1700k-1-3000_, 1.90 Å (Fig. 6 ▸ *a*). These results show that doses greater than 210 kGy did not contribute to a further improvement in resolution.

The global and local damage was inspected using the Wilson *B* factor (*B*_Wilson_; Table 2 ▸) and the *B* factor of S atoms (*B*_sulfur_; Supplementary Table S1), respectively. Table 2 ▸ and Supplementary Table S1 show that the *B*_Wilson_ and *B*_sulfur_ of D_21k-1-3000_ are larger than those of D_42k-2-3000,_ D_83k-13000_ and D_210k-1-3000_. We speculated that the large *B* of D_21k-1-3000_ was attributable to the quality of the data and is caused by the lower signal-to-noise ratio of D_21k-1-3000_. For this reason, the *B* factor of D_42k-2-3000_ is used as a standard. Fig. 6 ▸(*b*) shows *B*_0_/*B*_*n*_ as a function of dose following the work of Gotthard *et al.* (2019 ▸). Here, *B*_0_ denotes the *B*_Wilson_ or *B*_sulfur_ of D_42k-2-3000_ and *B*_*n*_ is that of D_83k-1-3000_ through D_1700k-1-3000_. The plot shows a nearly constant *B*_0_/*B*_*n*_ up to 210 kGy in both *B*_Wilson_ and *B*_sulfur_, indicating that the local and global damage was limited up to 210 kGy. The same tendency was also observed in our preliminary experiment conducted under almost the same conditions (Supplementary Fig. S7).

### 3.4. Effectiveness of the increase in the total number of merged images   

**Figure 7:**
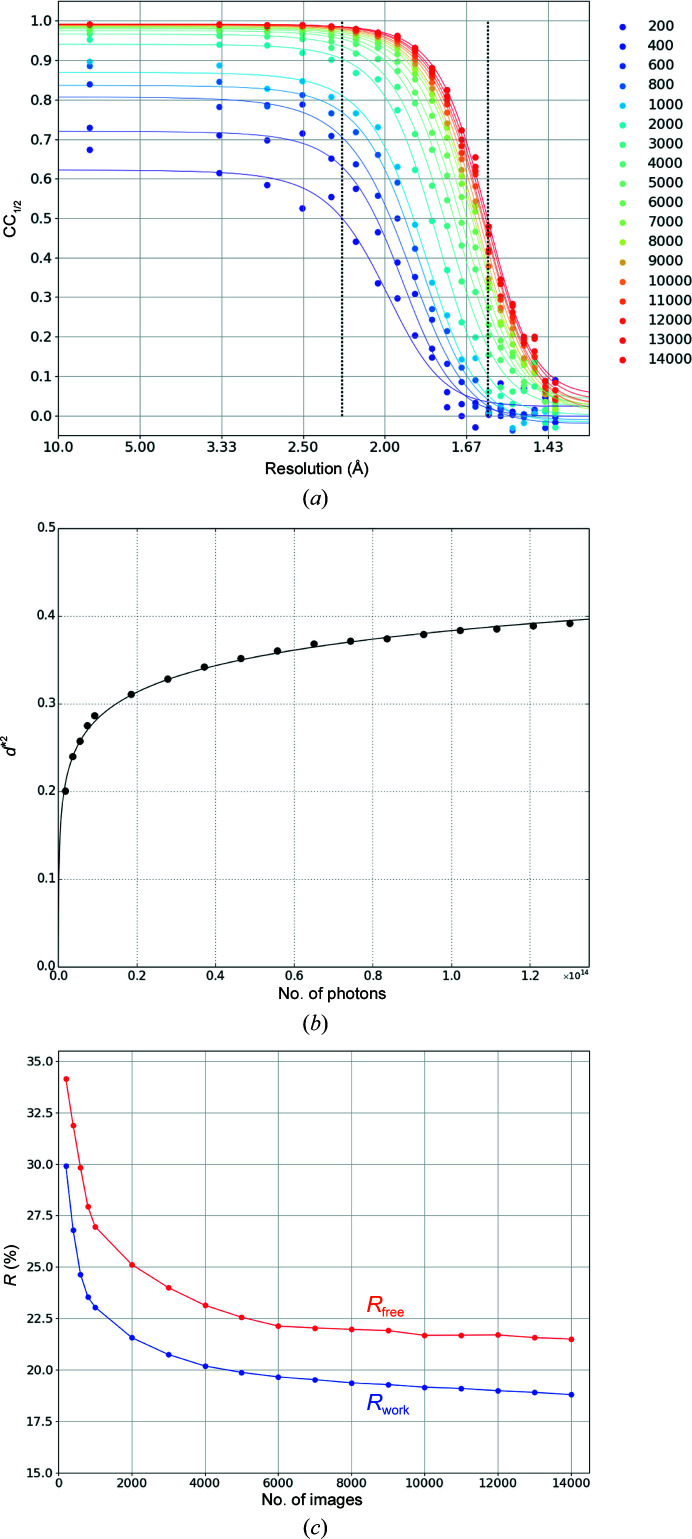
(*a*) CC_1/2_ of D_42k-*i*_ (*i* = 200, …, 14000) as a function of resolution together with a fitted line. (*b*) The square inverse of resolution, 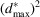, is plotted as a function of the total number of photons used to obtain the data sets. The solid line represents a fitted line using the logarithmic function explained in the main text. (*c*) *R*_free_ and *R*_work_ as a function of the number of merged images. All data sets were refined at 1.6 Å resolution.

The effectiveness of increasing the number of merged images was examined using 18 data sets D_42k-*i*_ prepared from L42k-1 and L42k-2, where *i* = 200, 400, … 1000, 2000, … 14000 denotes the number of merged images. The CC_1/2_ versus resolution plot indicates that the resolution increased from 2.23 to 1.60 Å between D_42k-200_ and D_42k-14000_ (Fig. 7 ▸ *a*). The square inverse of resolution, 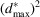, is shown in Fig. 7 ▸(*b*) as a function of the total number of photons used to obtain each data set, assuming that each image was obtained by illuminating a crystal with 9.3 × 10^9^ photons, which was calculated from the incident photon flux, the transmission of the filter and the exposure time. This figure corresponds to Fig. 2 of Yamamoto *et al.* (2017 ▸), showing that the 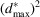 of a single thaumatin crystal increased as a function of the number of photons. Our results show that 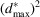 continued to increase after the initial rapid increase to 0.25 Å^−2^. The plot is well fitted by the logarithmic function 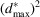 = *a* × ln(*b* × *N*_photon_), where *a* = 0.44 and *b* = 6.5 × 10^−11^. The significance of the increased resolution on merging was verified by *R*_free_ and *R*_work_ after refinement at 1.6 Å resolution (Fig. 7 ▸ *c*), which shows a decrease in *R*_free_ as a function of the number of merged images, indicating that an increase in the resolution was relevant for the structural analysis.

## 4. Discussion   

In this study, we have developed HAG SS-ROX and used it to evaluate a data-collection strategy for room-temperature micro-crystallography. The uniqueness of our method for RT SSX is the coating of crystals with PVA, which can protect them from changes in the environment surrounding the crystals (Baba *et al.*, 2013 ▸). The contribution of PVA to the background scattering was marginal. Although the PEEK film that stabilizes the humidity-controlled air made a significant contribution to increasing the background, it is not the only way to stabilize the air stream; we can also use a nozzle extension made of polyacetal that has a window of 5 mm in diameter through which X-rays can pass (see Fig. 1 in Baba *et al.*, 2019 ▸). The use of this extension could eliminate the influence of the PEEK film.

In HAG SS-ROX, data were collected by 2D raster scanning with rotation of the goniometer. In the best case, 9548 indexed images were obtained from crystals loaded onto a single mesh-loop of 2 × 2 mm in size (L42k-2). Merging 9000 images from them led to a resolution of 1.63 Å (D_42k-2_9000_) without significant radiation damage, which was confirmed by comparing structures obtained at various doses. The data collection took only 10 min, and the sample consumption was 0.25 µl of a 4 × 10^7^ ml^−1^ microcrystal suspension. Therefore, HAG SS-ROX can be said to be an efficient data-collection method. This efficiency comes from the high hit rate and index rate enabled by sample mounting using a mesh-loop, even though some improvements are needed to increase the reproducibility of a high hit rate (Table 1 ▸). The rotation of the goniometer might also have contributed to this high efficiency, as has been demonstrated in our previous work (Hasegawa *et al.*, 2017 ▸).

Wierman and coworkers have demonstrated the performance of serial oscillation crystallography with a fixed target, in which diffraction images were collected with an oscillation range of 1–5° from each crystal trapped in a well of silicon substrate (Wierman *et al.*, 2019 ▸). In their study using lysozyme crystals, a structure at 1.839 Å resolution was obtained from 95 data sets, where each data set was collected from a single crystal using a total oscillation of 3° with an angular step of 0.2°. In our experiment, 2000 crystals were needed to obtain a data set to 1.8 Å resolution, assuming that one image is obtained from one crystal (Fig. 7 ▸ *b*). Although direct comparison is difficult because the crystal size of 40 × 40 × 40 µm in the study of Wierman and coworkers is larger than that in our experiment, the reason why a more than tenfold larger number of crystals were needed in our case was partly attributed to the use of Monte Carlo integration. On the other hand, the total rotation range (the rotation angle per crystal multiplied by the total number of images) of 500° in our study is of the same order as the 285° in the study of Wierman and coworkers, meaning that the coverage of reciprocal space, which is related to the multiplicity of the data, is of the same order for both data-collection methods.

The comparison of data obtained at various absorbed doses revealed that an absorbed dose of up to 210 kGy was tolerable for both global and local damage, which was consistent with previous reports on global damage at RT (Nave & Garman, 2005 ▸; Southworth-Davies *et al.*, 2007 ▸) and with the observation of the local damage to the disulfide bond in thaumatin (Schubert *et al.*, 2016 ▸).

Although these dose limits exist, the achievable resolution can be improved by merging a number of images (Fig. 7 ▸). We found that the 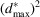 versus *N*_photon_ plot is well fitted with a logarithmic function: 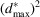 = 0.044 × ln(6.5 × 10^−11^ × *N*_photon_). To consider the meaning of this equation, we analytically derived the following equation starting from the well known equation for the Wilson plot (Wilson, 1942 ▸) as described in Appendix *A* [App appa],
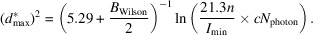



Here, *I*_min_ is the minimum intensity that can be measured as a signal, *n* is the number of atoms in the unit cell and *c* is a proportionality constant for *N*_photon_. This equation is less valid at high resolution due to the approximation of the squared atomic scattering factor by a single exponential function (Supplementary Fig. S8*a*). However, it provides a physical meaning for our experimentally derived equation. (i) It relates *I*_min_ to 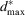 and indicates the importance of decreasing *I*_min_ to improve the resolution, which can be achieved by reducing the background scattering noise or systematic error in the measurement system, or by increasing the detector sensitivity. (ii) The equation also relates 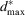 to *B*_Wilson_, meaning that the reduction of static or dynamic disorder in the crystal is effective for resolution improvement. The *B*_Wilson_ calculated by the equation is 35.3 Å^2^, which is twice as large as that derived from the Wilson plot (Table 2 ▸). This might be caused by an approximation introduced while deriving the equation, and some improvement is needed to use it for more quantitative analysis. (iii) The equation shows that 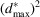 becomes 0 when *N*_photon_ is *I*_min_/21.3*nc*, meaning that meaningful data cannot be obtained at a photon flux less than this. In the case of our data in Fig. 7 ▸(*b*), *I*_min_/21.3*nc* is 1.5 × 10^10^. From a practical aspect, the relationship between 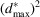 and *N*_photon_ could enable us to estimate the number of indexed images needed to achieve a desired resolution during SSX or SFX data collection.

In our study, a change in unit-cell dimensions during data collection was observed. A similar change was observed in the work of Tolstikova and coworkers in an SSX experiment using a 33 × 12 mm (horizontal × vertical) silicon chip enclosed in a measurement chamber into which humidity-controlled air was blown (Tolstikova *et al.*, 2019 ▸). Their results showed that there was a gradual change in unit-cell dimensions along the chip reflecting the humidity gradient in the measurement chamber. In our case, a humidity gradient would not be a problem considering the small mesh-loop size of 2 × 2 mm. One reason for the change in unit-cell dimensions is that data collection was started without waiting for an equilibrium between the humidity-controlled air and the vapor pressure of the PVA; *i.e.* evaporation of the PVA led to an increase in PVA concentration until the vapor pressure of the PVA reached the humidity of the surrounding air (73% relative humidity). This is supported by our previous work using a large single crystal, which showed that a decrease in humidity led to contraction of the *a* axis and elongation of the *c* axis (Baba *et al.*, 2013 ▸), as seen in this study. This change in unit-cell dimensions is mitigated by simply waiting for the equilibrium. However, a different tendency for change in unit-cell dimensions was observed (Supplementary Fig. S6), implying the presence of another cause. To make HAG SS-ROX more useful, further study is needed to stabilize the unit-cell dimensions. However, it is good news that non-isomorphous crystals can be discriminated, as demonstrated in Fig. 5 ▸(*b*).

## 5. Conclusion   

The importance of structure determination at RT has been rediscovered in recent years. Here, we have demonstrated that HAG SS-ROX is appliable to RT micro-crystallography and have evaluated an RT data-collection strategy. Our results have provided some insights into the influence of non-isomorphism, tolerable doses and the relationship between 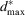 and *N*_photon_, all of which are practically useful in RT-SSX data collection. We also showed that the logarithmic function 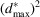 = *a* × ln(*b* × *N*_photon_) that was proposed on the basis of the experimental data could be analytically derived from the equation for the Wilson plot.

Thus far, we have demonstrated the usefulness of the HAG method as a tool to induce structural change by changing environmental parameters such as the temperature of a single-crystal data collection (Baba *et al.*, 2019 ▸). HAG SS-ROX enables us to extend the usefulness of the HAG method to micro-crystallography. Moreover, RT data collection using microcrystals paves the way for time-resolved SSX. We conclude that the establishment of HAG SS-ROX together with the strategy for RT data collection can contribute to the structural dynamics study of proteins on synchrotron beamlines.

## Supplementary Material

PDB reference: lysozyme, 7cdk

PDB reference: 7cdm

PDB reference: 7cdn

PDB reference: 7cdo

PDB reference: 7cdp

PDB reference: 7cdq

PDB reference: 7cdr

PDB reference: 7cds

PDB reference: 7cdt

PDB reference: 7cdu

Supplementary Figures and Table. DOI: 10.1107/S2059798321001686/nw5105sup1.pdf

## Figures and Tables

**Table 1:** Data-collection conditions, hit rates and index rates Measurements were made twice for each condition. A total of 22 200 images were collected for each data set.

Dose (kGy)	Dose rate (MGy s^−1^)	Data-set name	Transmission of filter (%)	Detector frame rate (Hz)	No. of hits	No. indexed	Hit rate (%)	Index rate (%)
21	2.1	L21k-1	9.49	100	8977	6715	40.4	74.8
		L21k-2			9581	7371	43.2	76.9
42	4.2	L42k-1	19.4	100	9617	7696	43.3	80.0
		L42k-2			13657	9548	61.5	69.9
83	8.3	L83k-1	40.1	100	12783	9240	57.6	72.3
		L83k-2			7987	6557	36.0	82.1
210	21	L210k-1	94.2	100	9803	5677	44.2	57.9
		L210k-2			5099	4003	23.0	78.5
420	23	L420k-1	100	54	12583	7615	56.7	60.5
		L420k-2			6404	4591	28.8	71.7
830	23	L830k-1	100	27	5081	3320	22.9	65.3
		L830k-2			15322	6886	69.0	44.9
1700	23	L1700-1	100	13	9856	5167	44.4	52.4
		L1700-2			3471	2691	15.6	77.5

**Table:** 

	D_42k-2_9000_	D_42k-2_bin1–5_	D_42k-2_bin6–10_	D_21k-1-3000_	D_42k-2-3000_
Data collection					
Resolution (Å)	55.45–1.70 (1.73–1.70)	55.47–1.70 (1.73–1.70)	55.42–1.70 (1.73–1.70)	55.42–1.80 (1.83–1.80)	55.42–1.80 (1.83–1.80)
No. of observed reflections	5115189 (170037)	2508284 (84880)	2608262 (85780)	1462471 (50222)	1530119 (53164)
No. of unique reflections	13805 (670)	13786 (677)	13831 (664)	11720 (577)	11720 (575)
Completeness (%)	100.0 (100.0)	100.0 (100.0)	100.0 (100.0)	100.0 (100.0)	100.0 (100.0)
Multiplicity	370.5 (253.8)	181.9 (125.4)	188.6 (129.2)	124.8 (87.0)	130.6 (92.5)
*R*_split_ (%)	9.87 (53.73)	13.99 (78.61)	14.08 (68.30)	17.59 (106.57)	15.95 (58.79)
〈*I*/σ(*I*)〉	8.32 (2.18)	5.87 (1.49)	5.99 (1.66)	4.82 (1.11)	5.50 (1.98)
CC_1/2_	0.9895 (0.7070)	0.9808 (0.5264)	0.9766 (0.5981)	0.9652 (0.4151)	0.9645 (0.6536)
Wilson *B* (Å^2^)	14.70	15.27	14.20	17.50	13.16
Refinement					
*R*_free_ (%)	21.07	22.74	21.49	22.71	21.65
*R*_work_ (%)	18.29	19.29	18.71	19.42	18.53
R.m.s.d., bond lengths (Å)	0.005	0.006	0.005	0.005	0.005
R.m.s.d., bond angles (°)	0.750	0.770	0.737	0.733	0.722
Ramachandran plot					
Favored (%)	98.43	99.21	98.43	99.21	99.21
Allowed (%)	1.57	0.79	1.57	0.79	0.79
Average *B* (Å^2^)					
Overall	15.87	16.35	15.36	17.69	13.96
Protein	15.35	15.86	14.85	17.24	13.45
Ligand	20.07	20.17	19.52	20.61	17.93
Water	25.93	25.85	25.36	26.49	23.93
Na^+^	13.31	13.66	13.18	16.40	12.04
PDB code	7cdn	7cdk	7cdm	7cdo	7cdp

**Table:** 

	D_83k-1-3000_	D_210k-1-3000_	D_420k-1-3000_	D_830k-2-3000_	D_1700k-1-3000_
Data collection					
Resolution (Å)	55.48–1.80 (1.83–1.80)	55.42–1.80 (1.83–1.80)	55.50–1.80 (1.83–1.80)	55.49–1.80 (1.83–1.80)	55.54–1.80 (1.83–1.80)
No. of observed reflections	1744998 (60363)	2136942 (74569)	1946241 (67386)	2257234 (77703)	2123889 (73299)
No. of unique reflections	11646 (575)	11698 (579)	11658 (575)	11677 (567)	11597 (568)
Completeness (%)	100.0 (100.0)	100.0 (100.0)	100.0 (100.0)	100.0 (100.0)	100.0 (100.0)
Multiplicity	149.8 (105.0)	182.7 (128.8)	166.9 (117.2)	193.3 (137.0)	183.1 (129.0)
*R*_split_ (%)	16.12 (58.78)	15.28 (60.27)	15.15 (73.84)	14.08 (86.38)	14.69 (168.43)
〈*I*/σ(*I*)〉	5.63 (2.06)	5.67 (1.97)	5.88 (1.81)	5.83 (1.37)	5.33 (0.78)
CC_1/2_	0.9552 (0.6948)	0.9711 (0.6723)	0.9669 (0.6241)	0.9789 (0.4599)	0.9774 (0.2465)
Wilson *B* (Å^2^)	13.13	13.60	15.46	19.02	23.18
Refinement					
*R*_free_ (%)	22.55	22.42	22.47	21.90	24.17
*R*_work_ (%)	18.98	18.75	18.88	19.08	20.26
R.m.s.d., bond lengths (Å)	0.006	0.006	0.005	0.006	0.005
R.m.s.d., bond angles (°)	0.776	0.757	0.752	0.776	0.744
Ramachandran plot					
Favored (%)	99.21	99.21	99.21	99.21	99.21
Allowed (%)	0.79	0.79	0.79	0.79	0.79
Average *B* (Å^2^)					
Overall	13.86	14.18	16.48	20.14	25.81
Protein	13.35	13.67	15.99	19.65	25.34
Ligand	18.94	18.18	21.13	26.20	33.10
Water	23.63	24.20	25.91	29.31	34.48
Na^+^	10.22	12.07	15.68	19.18	24.10
PDB code	7cdq	7cdr	7cds	7cdt	7cdu

## Appendix A Analytical derivation of the relationship between (*d**_max_)^2^ and *N*_photons_

The Wilson plot equation gives the relationship between the average observed intensity  and  as

where *f*_*j*_ is the atomic scattering factor and *n* is the number of atoms in the unit cell. *k* is a scaling factor, and can be substituted with *cN*_photon_, where *c* is a proportionality constant. Here, we introduce an approximation that expresses the squared atomic scattering factor as a single exponential:

The validity of this approximation is verified as follows: considering that the average numbers of atoms per amino-acid residue are five C atoms, 1.35 N atoms, 1.5 O atoms and eight H atoms, the average of the squared atomic scattering factor *f* ^2^_ave_ can be calculated as a weighted average of the *f* ^2^ of these atoms, where *f* can be expressed using the Cromer–Mann approximation. The least-squares fitting of (2) to *f* ^2^_ave_ in the *d** range 0–0.6 Å^−1^ gives *p* = 21.3 and *q* = 5.29, with a coefficient of determination *R* ^2^ of 0.99, indicating the soundness of this approximation in this resolution range (Supplementary Fig. S8*a*).

Use of this approximation in (1) leads to the equation

Defining *I*_min_ as the minimum intensity that can be measured as a signal, the achievable resolution  can be derived as follows, as illustrated in Supplementary Fig. S8(*b*),

which leads to
