# Supplementary material for: Evaluation of the data-collection strategy for room-temperature micro-crystallography studied by serial synchrotron rotation crystallography combined with the humid air and glue-coating method
Source: Acta Crystallogr D Struct Biol. 2021 Feb 25;77(Pt 3):300–12. doi: 10.1107/S2059798321001686 (PMC7919407; doi:10.1107/S2059798321001686)
Supplement: Supplementary file 1 [file d-77-00300-sup1.pdf]

Acta Cryst D
STRUCTURAL BIOLOGY

Volume 77 (2021)

Supporting information for article:

Evaluation of the data-collection strategy for room-temperature micro-crystallography studied by serial synchrotron rotation crystallography combined with the humid air and glue-coating method

Kazuya Hasegawa, Seiki Baba, Takashi Kawamura, Masaki Yamamoto and Takashi Kumasaka

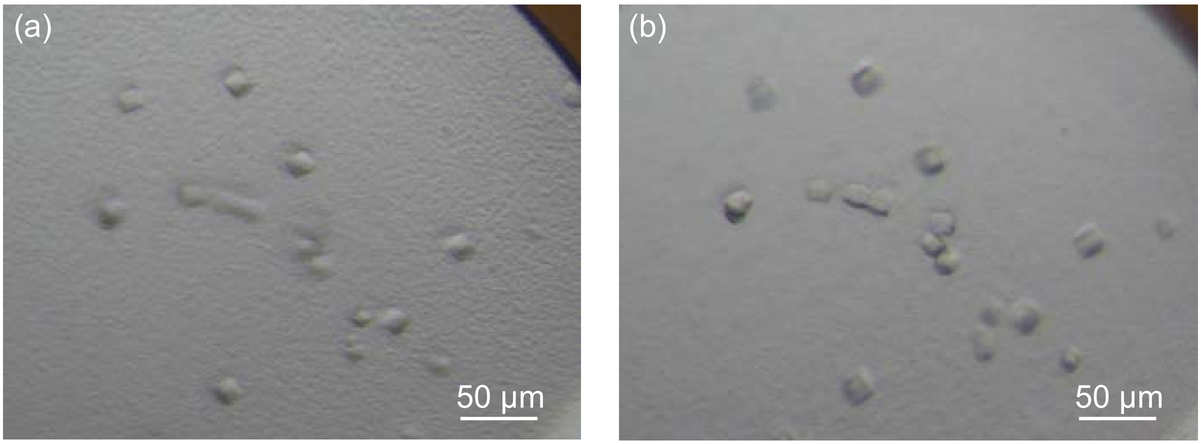

Figure S1 Observation of micro-crystals coated by PVA. In order to confirm that the micro-crystals were spontaneously coated by PVA, an experiment was conducted in the following steps; (1) PVA was spread over a round Litholoop (Protein Wave Corporation), and placed under humid-air of 73% RH, (2) suspension of lysozyme micro-crystals was deposited on the PVA, (3) waited for the evaporation of extra water in few minutes while keeping the Litholoop under the humid-air, (4) observed the surface roughness of PVA which reflected the formation of hydrogel in the presence of high concentration of salts, i.e. Na-Malonate, (5) confirmed the existence of crystals below the surface roughness of PVA. (a) Observation with focus at the surface of the PVA using Olympus SZX16 (objective lens SDFPLAPO2×PFC), (b) focus at micro-crystals at the center of image.

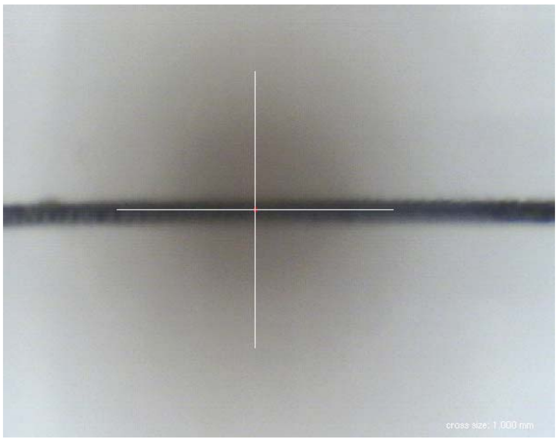

Figure S2 Side view of the mesh-loop observed by on axis microscope after data collection. The size of cross bar is 1 mm and red rectangle represents a beam size of 10 (V) × 8.7 (H) µm (FWHM).

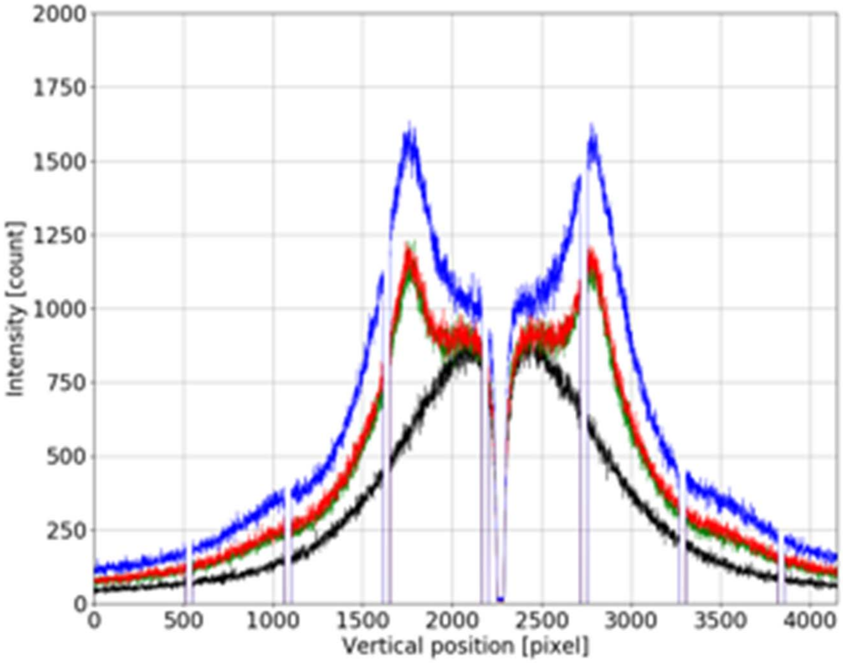

Figure S3 Comparison of the background profiles along the vertical direction of the scattering images. Black: air between the collimator and beamstop. Green: air and the PEEK film extending from the nozzle of the humidity blower. Red: air, the PEEK film and PVA spreading at the window position of the mesh-loop. Blue: air, the PEEK film, and polyimide of the mesh-loop coated with PVA. The absence of data in the interval of 530 pixels corresponds to the inter-module gap of the detector. The low intensity around 2272 pixel corresponds to the shadow of the beamstop.

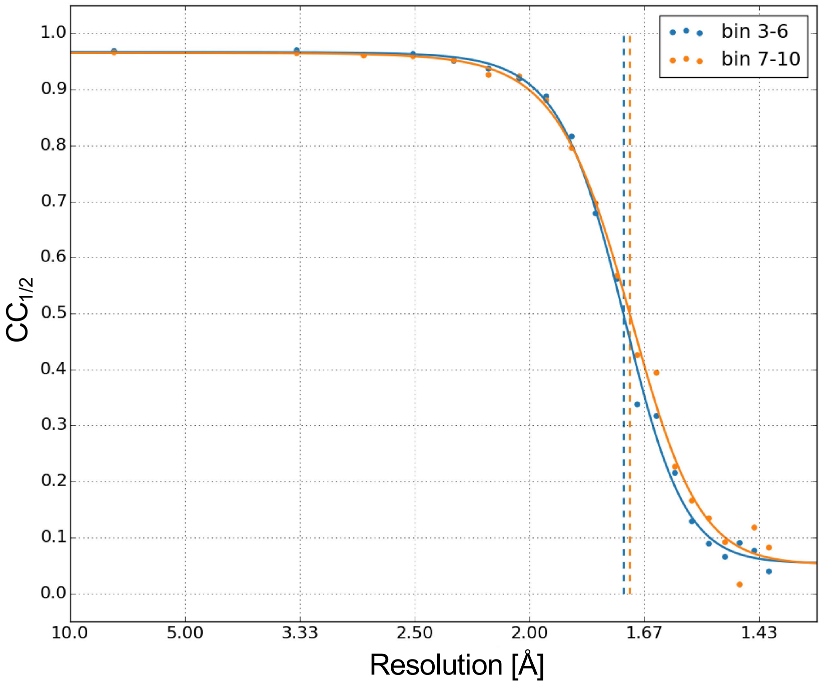

Figure S4 Comparison of $CC_{1/2}$ between two data sets prepared by merging 3500 images in bins 3–6 (sky blue) and 7–10 (orange) of L43k-2.

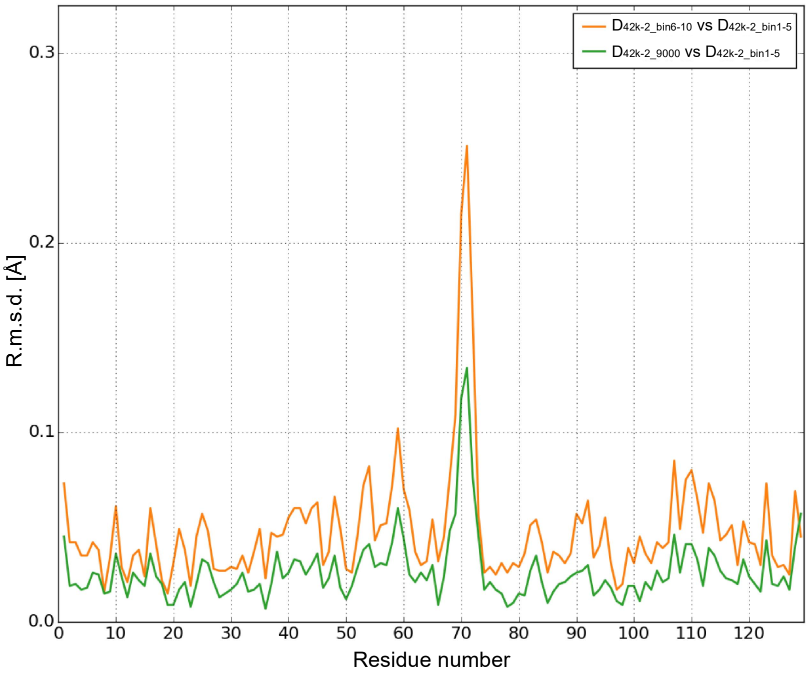

Figure S5 Main chain r.m.s.d. between $D_{42k\text{-}2\_bin1\text{-}5}$ and $D_{42k\text{-}2\_bin6\text{-}10}$ (orange) and between $D_{42k\text{-}2\_bin1\text{-}5}$ and $D_{42k\text{-}2\_9000}$ (green) as a function of residue number. The r.m.s.d between $D_{42k\text{-}2\_bin1\text{-}5}$ and $D_{42k\text{-}2\_9000}$ was smaller than that between $D_{42k\text{-}2\_bin1\text{-}5}$ and $D_{42k\text{-}2\_bin6\text{-}10}$, reflecting that the structure derived from $D_{42k\text{-}2\_9000}$ was the average structures of $D_{42k\text{-}2\_bin1\text{-}5}$ and $D_{42k\text{-}2\_bin6\text{-}10}$.

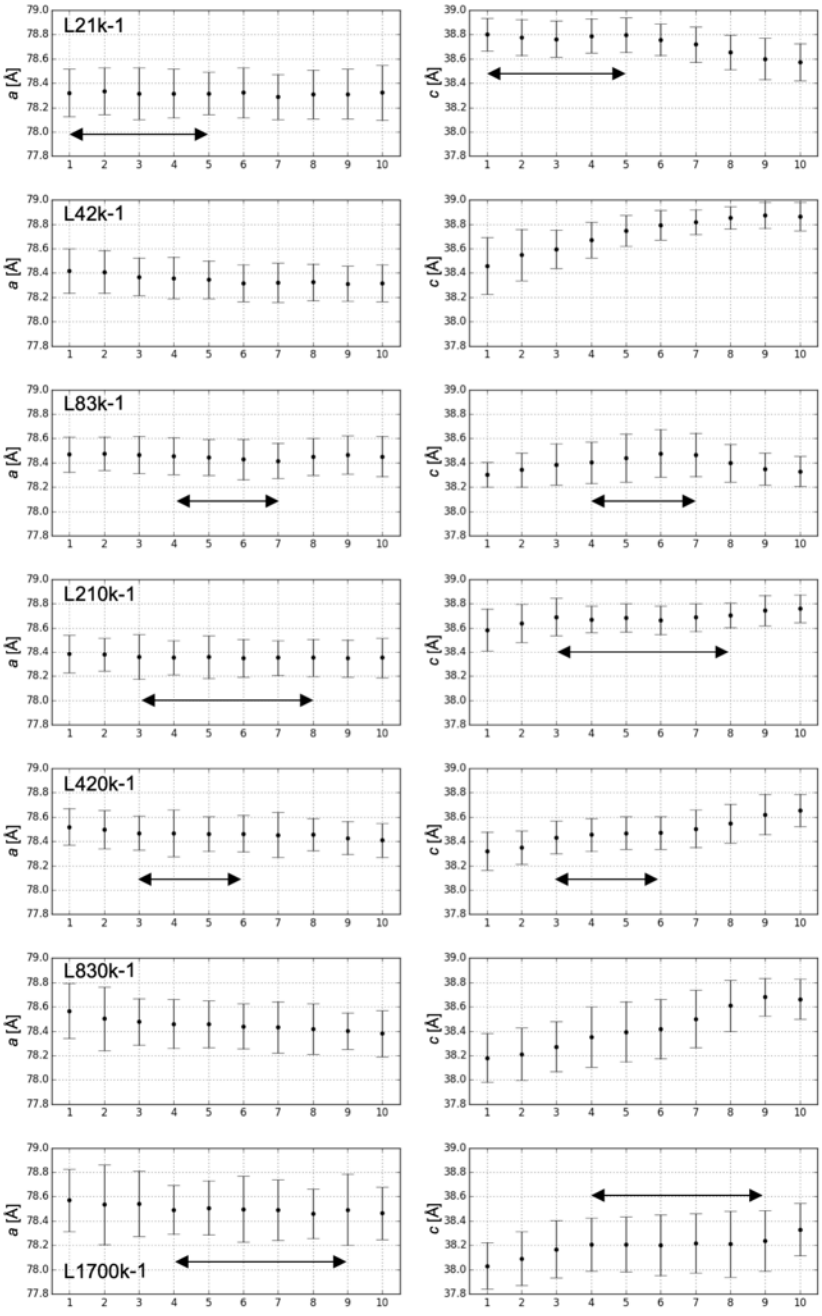

(a)

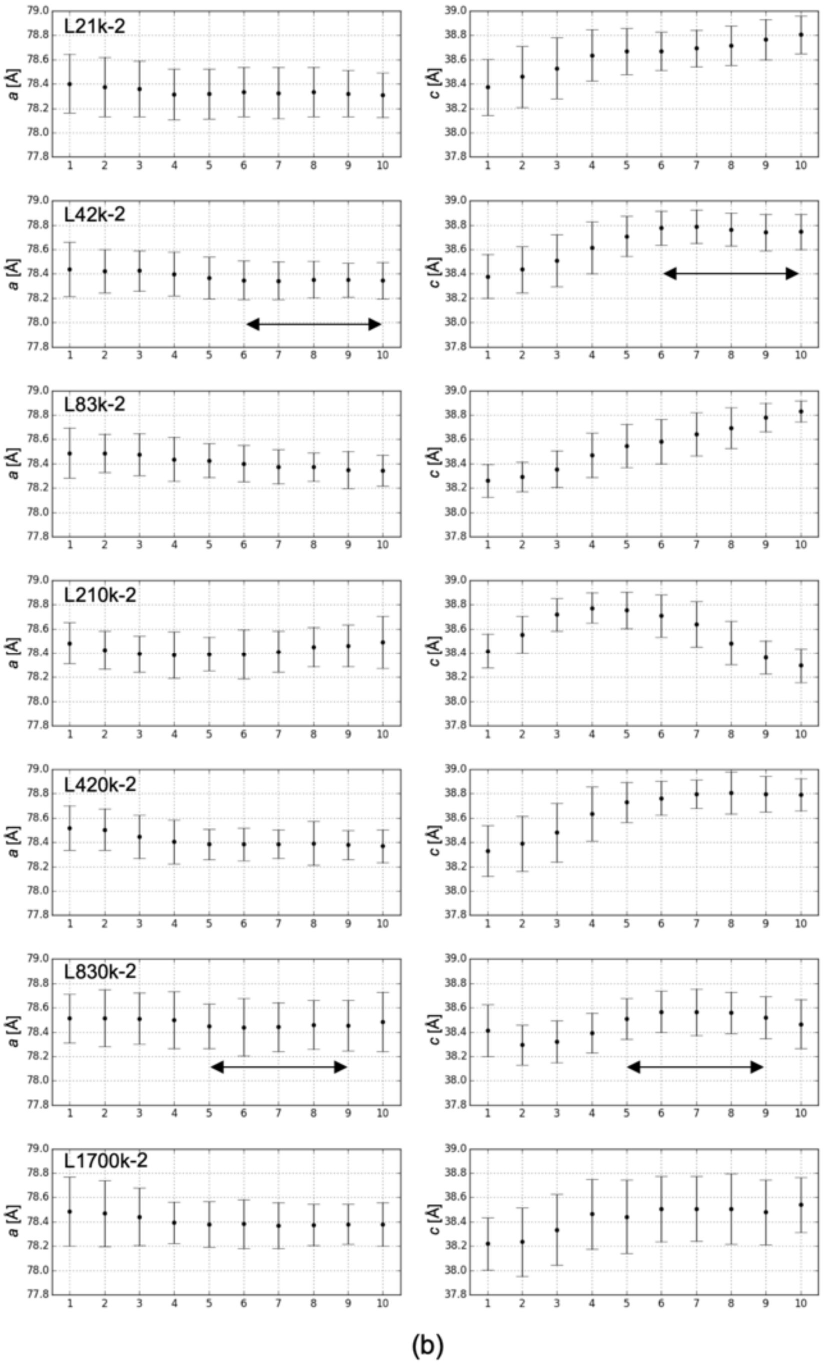

Figure S6 Change in cell dimensions during data collection for the (a) first series and (b) second series. The horizontal axis represents ten bins into which a total of 22,200 images were divided in sequential order. The vertical axis is the average cell dimensions of indexed images in each bin. The length of the *a* and *c* axes are shown in left and right panel, respectively. The error bar is ±σ. The two-

directional arrows indicate the bins used for preparation of the seven data sets to examine the influence of radiation damage, i.e. regions where the cell dimension change was small were selected to eliminate the influence of non-isomorphism.

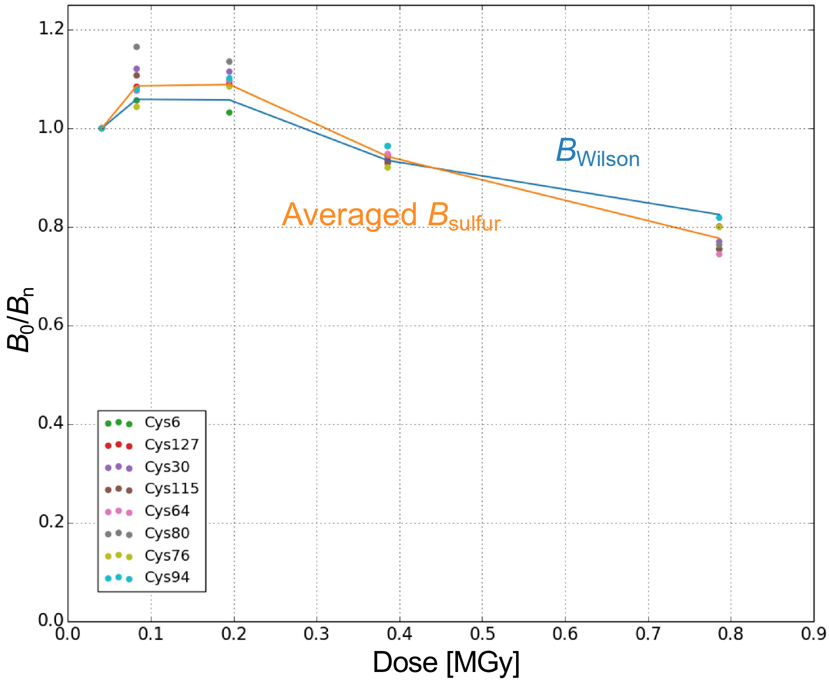

Figure S7 To confirm the reproducibility of the dose limit, B_0/B_n was plotted as a function of dose by using the result of our preliminary experiment in which data was collected with six dose, 41, 83, 195, 386 and 786 kGy. Data sets were prepared by merging 8000 images for each condition and structures were refined at 1.5 Å. The isomorphism was not checked here. B_0 denotes B_{Wilson} or B_{sulfur} of data set collected at 42 kGy. B_n is those of the other data sets. B_0/B_n of B_{Wilson} is shown as the solid sky blue line, and B_0/B_n of averaged B_{sulfur} of eight Cys Sγ is shown as the orange solid line.

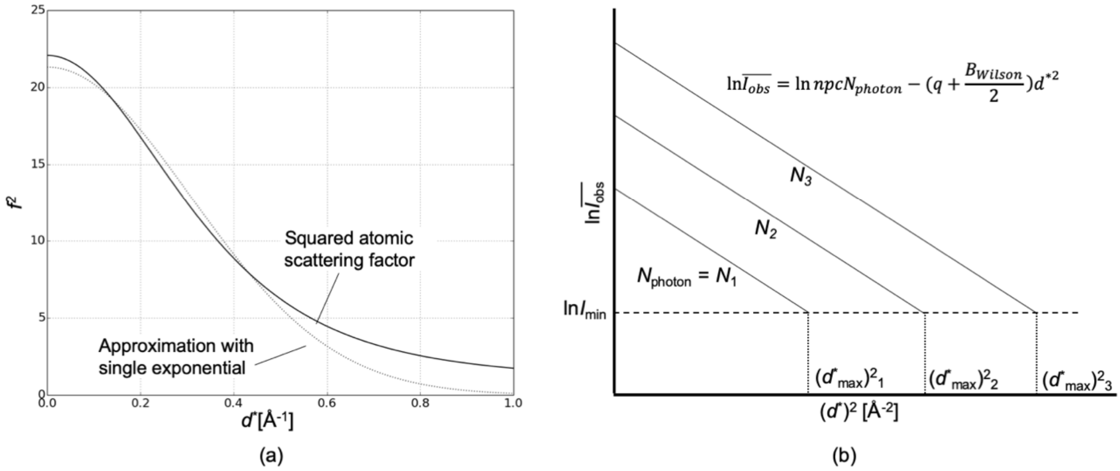

Figure S8 (a) Average of squared atomic scattering factor of C, N, O, and H atoms (solid line) and approximation using single exponential obtained by the least square fitting using the d^* range of 0 to 0.6 Å^{-1} (dotted line). (b) Schematic figure of relationship between I_{obs}, I_{min}, and d^*_{max}, illustrating that I_{obs} smaller than I_{min} cannot be observed as a meaningful signal, which limits the achievable resolution.

Table S1 *B* factor of *Sγ* of cysteine residues

Residue number	$D_{21k-1-3000}$	$D_{42k-2-3000}$	$D_{83k-1-3000}$	$D_{210k-1-3000}$	$D_{420k-1-3000}$	$D_{830k-2-3000}$	$D_{1700k-1-3000}$
6	19.5	16.2	15.3	16.4	19.6	22.7	29.9
127	18.6	14.5	14.2	16.1	18.2	22.2	28.7
30	11.4	7.3	8.5	8.1	10.4	14.1	21.1
115	10.8	6.9	7.0	6.6	9.1	12.8	18.3
64	12.1	7.3	6.7	7.8	9.5	13.8	19.4
80	13.1	8.6	7.3	8.2	9.9	14.7	20.3
76	16.9	13.7	12.7	12.8	16.4	19.7	25.4
94	14.4	10.6	10.7	10.9	12.4	18.5	23.6
